# Comprehensive Performance Evaluation for Hydrological and Nutrients Simulation Using the Hydrological Simulation Program–Fortran in a Mesoscale Monsoon Watershed, China

**DOI:** 10.3390/ijerph14121599

**Published:** 2017-12-19

**Authors:** Zhaofu Li, Chuan Luo, Kaixia Jiang, Rongrong Wan, Hengpeng Li

**Affiliations:** 1 College of Resources and Environmental Sciences, Nanjing Agricultural University, Nanjing 210095, China; lc_notek@163.com (C.L.); fly_kaixiajiang@163.com (K.J.); 2 Nanjing Institute of Geography and Limnology, Chinese Academy of Sciences, No. 73, Beijing East Road, Nanjing 210008, China; rrwan@niglas.ac.cn (R.W.); hpli@niglas.ac.cn (H.L.)

**Keywords:** HSPF, streamflow, nonpoint source pollution, Taihu Lake region

## Abstract

The Hydrological Simulation Program–Fortran (HSPF) is a hydrological and water quality computer model that was developed by the United States Environmental Protection Agency. Comprehensive performance evaluations were carried out for hydrological and nutrient simulation using the HSPF model in the Xitiaoxi watershed in China. Streamflow simulation was calibrated from 1 January 2002 to 31 December 2007 and then validated from 1 January 2008 to 31 December 2010 using daily observed data, and nutrient simulation was calibrated and validated using monthly observed data during the period from July 2009 to July 2010. These results of model performance evaluation showed that the streamflows were well simulated over the study period. The determination coefficient (*R*^2^) was 0.87, 0.77 and 0.63, and the Nash-Sutcliffe coefficient of efficiency (Ens) was 0.82, 0.76 and 0.65 for the streamflow simulation in annual, monthly and daily time-steps, respectively. Although limited to monthly observed data, satisfactory performance was still achieved during the quantitative evaluation for nutrients. The *R*^2^ was 0.73, 0.82 and 0.92, and the Ens was 0.67, 0.74 and 0.86 for nitrate, ammonium and orthophosphate simulation, respectively. Some issues may affect the application of HSPF were also discussed, such as input data quality, parameter values, etc. Overall, the HSPF model can be successfully used to describe streamflow and nutrients transport in the mesoscale watershed located in the East Asian monsoon climate area. This study is expected to serve as a comprehensive and systematic documentation of understanding the HSPF model for wide application and avoiding possible misuses.

## 1. Introduction

Human activities are accelerating environmental changes throughout the world, including threatening freshwater resources and aquatic biodiversity [1,2]. Eutrophication as a result of human activities is a widespread problem in rivers, lakes, estuaries, and coastal oceans that is caused by nutrient enrichment, which seriously degrades aquatic ecosystems and impairs the use of water for drinking, industry, agriculture, recreation, and other purposes [3]. In recent years, many countries have confirmed that nonpoint source pollution coming from agriculture and urban activities in the watershed scale are major sources of nitrogen and phosphorus to aquatic ecosystems [3]. In the watershed scale, nonpoint source pollution is related to rainfall runoff, soil erosion and chemical substance migration. The occurrence of nutrient transport is driven by the hydrological processes, which are mainly affected by factors such as climate, soil, vegetation, land use topography, etc. These factors have obvious spatial and temporal variability. Monitoring alone is not enough to explain the variation of water quality owing to system complexity and various anthropogenic impacts [4]. Therefore, the most effective method for the quantitative study of nonpoint source pollution is to build a model to simulate these processes in different spatial scales and temporal series. Modeling has become one of the most powerful tools for better understanding the laws of these processes and taking effective measures to control or mitigate nonpoint source pollution.

Since the 1970s, several hydrological and water quality models have been developed to assist in understanding hydrologic systems and pollutant loadings [5]. These models include simple empirical statistical models, such as Universal Soil Loss Equation (USLE) [6], and complex physical and chemical process models, such as Chemicals, Runoff, and Erosion from Agricultural Management Systems (CREAMS) [7] and Areal Non-point Source Watershed Environment Response Simulation (ANSWERS) [8]. Furthermore, many models enhanced their simulation capability and maneuverability by integrating with geographical information systems (GIS) and graphical user interfaces, such as Annualized Agricultural Non-Point Source (AnnAGNPS) [9], Soil and Water Assessment Tool (SWAT) [10], Hydrological Simulation Program–Fortran (HSPF) [11], etc. The principal advantage of such a model is that it affords a realistic representation of the spatial variability of watershed characteristics [12]. These models can be used to simulate the transportation processes of runoff, sediment, nutrient and other chemical substances. Detailed reviews of these models can be found in the literature [13,14]. It is worth noting that good hydrological simulation is the basis of water quality modeling. Among many integrated models currently available, the HSPF is one of the best models due to its favorable performance of hydrological process simulation. HSPF evolved from the Stanford Watershed Model (SWM), the Agricultural Runoff Management (ARM) model, and the Nonpoint Source (NPS) model [12]. As a comprehensive model, HSPF was developed by the United States Environmental Protection Agency (USEPA) for simulating many processes related to water quantity and quality in watersheds [15], and it has been used worldwide during the past decade [16].

Many studies have been carried out to simulate hydrological processes using HSPF around the world. Integrated with the paddy runoff model (PRM) and the lake discharge model (LDM), HSPF was used to simulate daily runoff in the middle and lower regions of the Changjiang (Yangtze River) basin [17]. On a monthly basis analysis, the results of a case study demonstrated that HSPF is capable of simulating hydrology in a river for a tropical island watershed [18]. In a Mediterranean temporary river basin, the seasonal variability of flow was captured by the combination of HSPF and the karstic spring flow model [19]. Hydrological processes in the Mobile River watershed located in the northern Gulf of Mexico, were modeled using HSPF with support of the Better Assessment Science Integrating point & Nonpoint Sources (BASINS) GIS system [20]. The simulated results are in a very good agreement with the observed field data for the simulation of surface and groundwater flow in a karstic river basin where flood phenomena appear from time to time in Greece [21]. From a case study in Hualien County, Taiwan, the results reveal that the HSPF model has a better performance than the FLO-2D model (a two-dimensional commercial model) at peak flow and flow recession period, and can successfully replicate the influence zone of the debris-flow disaster event with an acceptable error [22]. The HSPF model was successfully adapted to model daily streamflow processes in an upland basin in Alabama and Mississippi (Luxapallila Creek—1856 km^2^) and in a steep-slope tropical catchment in Puerto Rico (Rio Caonillas—99 km^2^) however, the model performance was poor in a humid subtropical watershed in coastal Alabama (Fish River—140 km^2^) [23].

As a comprehensive watershed model, in addition to simulating hydrology, HSPF has been widely used to simulate the transport processes of selected water quality constituents, including sediment and nutrients by calculating mass balance. The simulations of nitrogen and phosphorus concentrations during a first flood event have been performed using the HSPF model in the Iskar River (Bulgaria) [24]. The HSPF was used to simulate the runoff and nonpoint source pollution load from a mountainous forest watershed in Taiwan, China [25]. The land use effects on fluxes of suspended sediment, nitrogen and phosphorus from a river catchment of the Great Barrier Reef, Australia was studied using HSPF [26]. Research results indicate that the HSPF model can be used as a tool for simulating runoff and sediment associated NPS pollution losses from a small mixed type watershed of the Damodar Valley Corporation (Hazaribagh, India) [27,28]. The HSPF model was effective in describing pollutant behavior in the 51 km^2^ Nogok stream watershed in Korea [29]. The HSPF was used to assess the effects of land uses on river water quality in the Mun River basin (Thailand) [30]. The HSPF model was used to predict soil erosion and sediment yield from typical watersheds in Hualien County on the eastern coast of Taiwan [31] and in the Tahan river watershed in northern Taiwan [32]. The daily nitrate-nitrogen concentrations were simulated using HSPF for the Amite River in Louisiana, USA [33]. Quantitative estimation of the nonpoint source load by HSPF was performed in the Dongcheon estuary, Korea [34]. Nutrient dynamics during flood events were simulated in a tropical catchment in Southern Vietnam [4]. HSPF was employed to simulate the nutrient export from a small hilly watershed in an eastern monsoon region of China [35].

Through the above studies, HSPF has been widely used around the world and presents a good performance in simulating various hydrological processes under different rainfall conditions and modeling transport processes of water quality components. However, the models must be thoroughly tested by applying them to various watersheds before using them in management decisions [27]. It can be predicted that research on and applications of the HSPF model will be more and more widely reported in the future. On the one hand, the research on the development and improvement of the HSPF model will continue, including model calibration method research [36], model platform development [37], model function expansion [38], parameter estimation and uncertainty research [39,40] and so on. On the other hand, the application of the HSPF model will be more extensive, in different countries and regions, focusing on climate change, land use change and other natural processes or human activities impact on hydrology and water quality, for the purpose of resolving the problems of the integrative management for water resources and water environments in watershed or region scale. Thus, it is important to have a clear understanding of simulation capabilities of the model for its appropriate use and avoiding possible misuses [27].

As one of the most densely populated region, the rapid development of the economy along with a large demand for clean water, as well as a large amount of domestic and industrial sewage discharge, the Taihu Lake basin is one of areas with the most serious water environmental problems, which has become an important factor restricting regional development in China [41]. One of the main tributaries of Taihu Lake, the Xitiaoxi River watershed, is taken as the study area, which is located in the subtropical monsoon region with a hilly landform, dominated by agriculture and forestry land. It is a representative area of HSPF model application in China.

The purpose of this study is to construct and evaluate the performance of the HSPF model for hydrological and nutrient simulation from the mesoscale monsoon watersheds in China. The detailed objectives of this study are to: (1) construct the HSPF model in the Xitiaoxi River watershed, including data acquisition, parameter adjustment, calibration and validation, etc.; (2) simulate the daily, monthly and annual runoff of the watershed, and evaluate the model performance; and (3) analyze and evaluate the results and performance of nitrogen and phosphorus nutrient simulation. This study is a comprehensive and systematic examination of the HSPF model, which has an important reference value for the hydrological and water quality simulations.

## 2. Materials and Methods

### 2.1. Study Area

**Figure 1:**
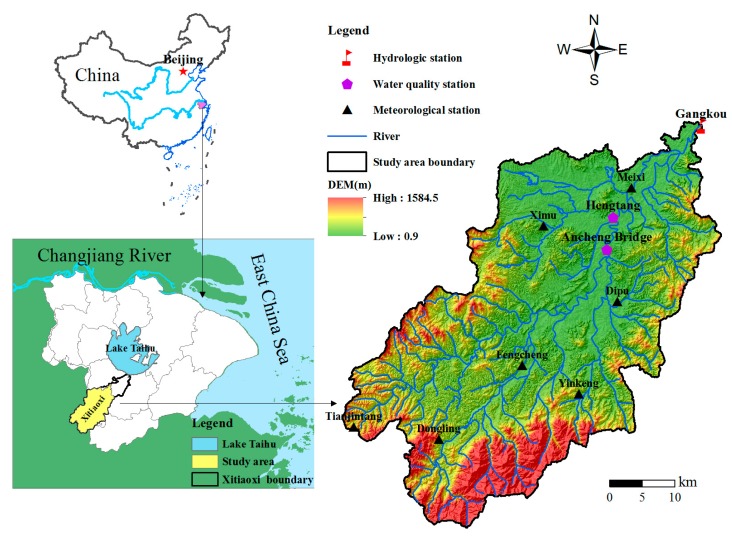
Location of the Xitiaoxi watershed and distribution of hydrologic, water quality, meteorological stations.

The Xitiaoxi River watershed is located at 119°14’–119°45’ E and 30°22’–30°45’ N, southwest of the Taihu Lake basin (Figure 1). Known also as the Western Tiaoxi River, the Xitiaoxi River merges with the Eastern Tiaoxi River to flow into Taihu Lake. As one of the most important tributaries, its main stream length is 159 km, and it contributes a large proportion of runoff and nutrient load to Taihu Lake [42]. Along the direction of the river from southwest to northeast, the Xitiaoxi watershed contains three different geomorphic regions. Mountainous areas are distributed in the southwest part of the upper reaches, with an elevation over 600 m and maximum elevation of 1587.4 m. Hilly areas are located in the center with an elevation of 150–600 m. A flat alluvial plain lies in the northeastern part with a low hydraulic gradient and well developed drainage network [43,44].

The watershed is characterized by a subtropical monsoon climate with an average annual temperature of 15.5 °C and an average annual precipitation of 1465.8 mm [42]. The spatio-temporal variations in the precipitation and evaporation distribution are statistically significant [43]. The annual rainfall gradually decreased from 1800 mm in the southwest mountain area to 1200 mm in the northeast plains [43]. More than 75% of the rainfall occurs in the wet season from April to October, while less than 25% occurs in the dry season from December to March [45]. The drainage pattern in the Xitiaoxi basin is of dendritic type [43]. The distribution of surface runoff in the catchment is mainly controlled by rainfall and land cover, so the variety of surface runoff in the Xitiaoxi catchment is seasonal, and the annual change of surface runoff is distinct [46].

The predominant soil type is yellowish red earth which accounts for 49.2% of the study area, followed by paddy soil and yellow earth which account for 21.2% and 11.5%, respectively [47]. Although the watershed is predominantly rural, land use in this watershed is diversiform, of which approximately 65% and 26.3% was covered by forest land and agriculture land, respectively. The remaining area was covered by garden land, urban or built-up, grass and wetlands/water. See Figure 1 for a map of the study area.

### 2.2. HSPF Modeling Approach

#### 2.2.1. Description of HSPF

HSPF is a comprehensive watershed model that simulates non-point sources of runoff and pollutant loadings for a watershed, combines these with point sources contributions, and models flow and water quality transport and fate [48]. The HSPF model was built in Better Assessment Science Integrating point and Nonpoint Sources (BASINS), which is a GIS-based multipurpose environmental analysis system developed by the USEPA [48]. Users can divide the study watershed into many sub-watersheds through the BASINS system, and then turn to WinHSPF. WinHSPF is an interactive windows interface, which consists of three application modules: Pervious land (PERLND), Impervious land (IMPLND) and Reach & Reservoir (RCHRES). PERLND and IMPLND modules are used to simulate the hydrologic and water quality processes over pervious and impervious land surfaces, respectively. The RCHRES module was developed to represent hydrologic and water quality processes for streams and well-mixed impoundments. Water balance for selected points can be calculated by HSPF based on inputs of precipitation, with hydrological parameters for different land cover classes. Associated water quality such as nutrients are modeled by using a system of coupled mass balance equations describing each nutrient compartment and each of the following constituents: dissolved inorganic and organic nutrients, particulate organic nutrients and sediment nutrients. Detailed information about the structure and theory of HSPF can be found in the HSPF version 12.2 user’s manual [48].

#### 2.2.2. Data Acquisition for HSPF Model Construction in the Xitiaoxi Watershed

HSPF requires extensive data input and complex procedure in the initial stage. Required input data includes a digital elevation model (DEM), land use data, soil data and meteorological data. For the Xitiaoxi watershed, the DEM with a 30 × 30 m horizontal resolution was extracted from a 1:50,000 relief map. Land use data with 30 m grid size were interpreted based on SPOT imagery acquired in 2004. The soil type map with a 1:600,000 scale was generated in 2000 by the local soil survey department. The meteorological data for the simulation (2002–2010) were collected from seven meteorological stations (Figure 1) distributed in the Xitiaoxi watershed. Eight meteorological elements include solar radiation, air temperature, dew-point temperature, precipitation, evapotranspiration, wind speed, cloud cover, and atmospheric pressure were acquired for running HSPF model.

In addition to the abovementioned data, HSPF also required hydrological and water quality time series data for model calibration and validation. The daily observed flow data for the period from 1 January 2002 to 31 December 2010 were collected from the Gangkou hydrological station located at the watershed outlet. The monthly observed nutrient data for the period from July of 2009 to July of 2010 were obtained from the Hengtang and Ancheng Bridge water quality monitor stations. The locations of the hydrological and water quality stations are shown in Figure 1.

#### 2.2.3. Model Calibration and Validation

Model calibration and validation are essential steps for improving the quality of the model simulation. Calibration is a process of adjusting model parameters within a suitable range to achieve agreement between observed and simulated data, while model validation is a process of evaluating the calibrated model parameters to determine the most matching model parameters [49]. Calibration of the HSPF model is an iterative process that is used to establish the most suitable values for process-related parameters [35]. In this research, reviewed by literature of HSPF application around the world and considering the experiences in the similar watershed in Taihu Basin [35], especially drawn on the results of parameter sensitivity analysis in the watershed [40], a total of nine parameters for runoff and twelve parameters for nutrients were confirmed for adjustment during the calibration period (Table 1).

Runoff simulation was calibrated by integrating with the Model-Independent Parameter Estimation Tool (PEST) program [50], which is a nonlinear optimization technique and has been widely applied for HSPF hydrology calibration [51,52]. The selected runoff parameters were input into the PEST program and made PEST run, and the running process was an iterative operation until the time series of simulated and observed runoff matched the best. Validation was performed by comparing the matching degree of simulated and observed values based on the results of calibration. The calibration for runoff was performed from 1 January 2002 to 31 December 2007 and then validated from 1 January 2008 to 31 December 2010 at the Gangkou hydrological station.

Moreover, nutrients concentration were manually calibrated at the Hengtang station during July 2009–July 2010. The parameters were repeatedly adjusted for nutrient components to make the simulated values approximate the observed ones as much as possible, and validation was performed at the Ancheng Bridge station for the same period as calibration. The calibrated values of hydrological and nutrient parameters are list in Table 1.

#### 2.2.4. HSPF Model Performance Evaluation

The results of the simulation were analyzed for “goodness-of-fit” with observed data. The performance evaluation of HSPF was carried out in a comprehensive manner, as suggested by previous research [53,54]. The coefficient of determination (*R*^2^), Nash-Sutcliffe coefficient of efficiency (Ens) [55], revised Ens (Ens’) and the percent bias (PBIAS) were employed for model assessment. The coefficient of determination (*R*^2^) is a number that indicates the proportion of the variance in the dependent variable that is predictable from the independent variable(s). It ranges from 0 to 1, with higher values indicating better agreement. The Ens indicates the consistency with which simulated values versus observed values follow a best-fit line. Van et al. [56] proposed results as being considered highly satisfactory for an Ens value equal to or larger than 0.75, satisfactory between 0.36 and 0.75, and unsatisfactory for Ens smaller than 0.36. It should be noted that *R*^2^ and Ens are oversensitive to extreme values, which may mislead the evaluation of model performance. To avoid this, a revised Ens was defined as Ens’, which could reduce the effect of squared terms [53]. In general, Ens’ has a lower value than Ens, and when Ens’ ranges from 0.51 to 0.71, the model can be considered satisfactory [57]. Percent bias (PBIAS) measures the average tendency of the simulated data to be larger or smaller than their observed counterparts. Model efficiencies were classified by Moriasi et al. [54] and Parajuli et al. [58], as being considered excellent when values inferior to 10% and inferior to 25% for runoff and nutrients prediction, respectively. They are considered good when values range from 11% to 25% and 26% to 40% for runoff and nutrients prediction, respectively. The formulas for these coefficients are as follows:

(1) $$R^{2}=\frac{{\left[\sum_{i=1}^{n}\left(S_{i}-S\right)\left(O_{i}-O\right)\right]}^{2}}{\sum_{i=1}^{n}{\left(S_{i}-S\right)}^{2}\sum_{i=1}^{n}{\left(O_{i}-O\right)}^{2}}$$

(2) $$\mathrm{Ens}\;=\;1-\frac{\sum_{i=1}^{n}{\left(S_{i}-O_{i}\right)}^{2}}{\sum_{i=1}^{n}{\left(O_{i}-\;O\right)}^{2}}$$

(3) $${\mathrm{Ens}}^{\prime }=\;1-\frac{\sum_{i=1}^{n}\left|S_{i}-O_{i}\right|}{\sum_{i=1}^{n}\left|O_{i}-\;O\right|}$$

(4) $$\mathrm{PBIAS}\;=\;\frac{\sum_{i=1}^{n}\left(O_{i}-S_{i}\right)}{\sum_{i=1}^{n}O_{i}}\times 100$$

where $S_{i}\;$is the simulated data, $O_{i}$ is the observed data, *S* is the mean of simulated data set, *O* is the mean of the observed data set, i is the ith event, and n is the number of observations.

## 3. Results and Analysis

### 3.1. Hydrological Simulation Results in Multiple Time Steps and Model Performance

#### 3.1.1. Annual Streamflow Simulation Results and Model Performance

**Figure 2:**
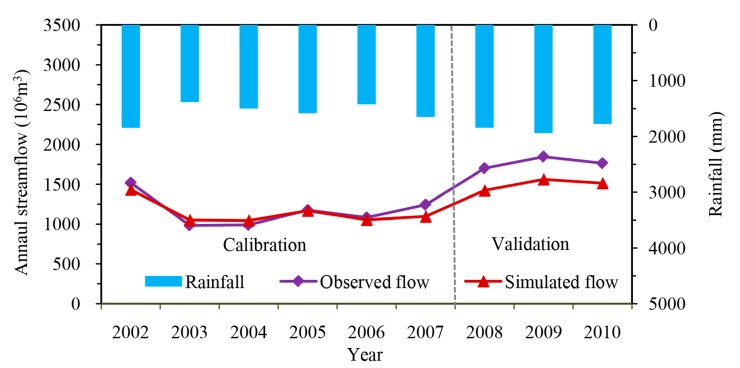
Comparison of simulated and observed annual streamflow in the Xitiaoxi watershed during the calibration and validation period.

The simulated annual streamflow was plotted with the observed data during the calibration (2002–2007) and validation (2008–2010) periods (Figure 2). There were generally good agreements between the simulated and observed annual streamflow across the entire simulation period. During the calibration period (2002–2007), the observed annual streamflow ranged from 984.0 × 10^6^ m^3^ to 1518.9 × 10^6^ m^3^ in 2003 and 2002 respectively, corresponding with the rainfalls of 1371.4 mm and 1834.9 mm, respectively. The simulated annual streamflow in 2003 and 2002 was 1050.8 × 10^6^ m^3^ and 1432.8 × 10^6^ m^3^, which is somewhat overestimated and underestimated compared with the observed value, respectively. The relative error (RE), which is a deviation percentage between simulation and observation values, ranged from −11.8% to 6.8% with an average value of −2.1% for the calibration years. During the validation period (2008–2010), the observed annual streamflow ranged from 1700.3 to 1845.9 in 2008 and 2009, corresponding with the rainfall of 1831.7 mm and 1926.3 mm, respectively. The simulated annual streamflow in corresponding years was 1432.5 × 10^6^ m^3^ and 1561.9 × 10^6^ m^3^, and there is a certain amount of underestimation compared with the observed value. The RE ranged from −14.2% to −16.3%, with an average of −15.3%.

Statistical indexes (Table 2) of model performance shown quantitatively with the consistency between simulated and observed annual streamflow. During the calibration period, the annual streamflow hydrographs for the 6-year simulation period displayed commendable agreement and tendency, and the *R*^2^, Ens, Ens’ and PBIAS for annual streamflow simulation were 0.867, 0.816, 0.560 and 2.1%, respectively. During the validation period, the *R*^2^ is 0.940, whereas the PBIAS is 15.3%, which means the simulated values are in good agreement with the observed ones, but there is a certain amount of deviation for the streamflow. This deviation was further represented by the poor performance of Ens and Ens’, with values of −19.6 and −4.3, respectively. This may be attributed to the validation period being all wet years, and there is a certain amount of underestimation for streamflow simulation in wet years [49]. Nevertheless, these performance indexes also indicated that the HSPF model is very good in annual streamflow simulations in the Xitiaoxi watershed.

#### 3.1.2. Monthly Streamflow Simulation Results and Model Performance

**Figure 3:**
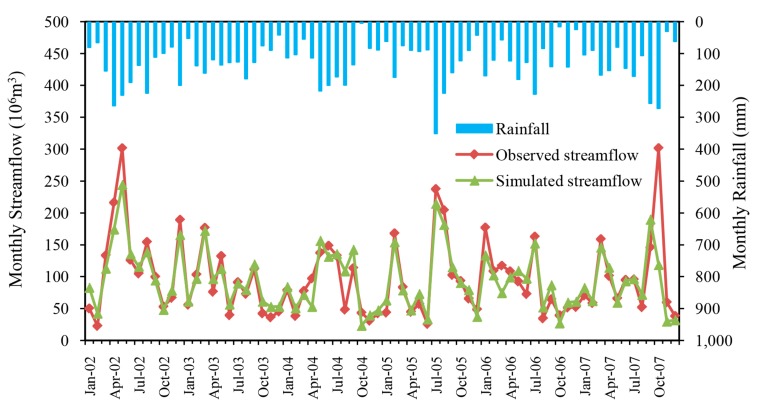
Comparison between simulated and observed monthly streamflow in the Xitiaoxi watershed during the calibration period (January 2002 to December 2007).

**Figure 4:**
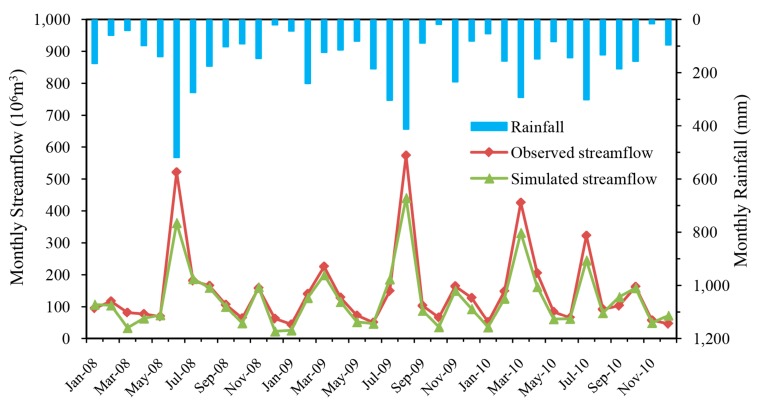
Comparison between simulated and observed monthly streamflow in the Xitiaoxi watershed during the validation period (January 2008 to December 2010).

The simulated and observed monthly streamflow at the Gangkou hydrologic station during calibration (January 2002 to December 2007) and validation (January 2008 to December 2010) periods are plotted in Figure 3 and Figure 4. As seen from the two hydrographs, the simulated monthly streamflow matched very well with observed values during the whole simulation period. It is obvious that the streamflow simulated by HSPF has a good response to rainfall. Consistent with the rainy season, the simulated and observed flood peak flows appeared in the same time. However, there is a certain underestimation of the simulated value compared with the measured value during the flood month, and this discrepancy may be caused by uncertainty associated with the rainfall databases, effects of spatial discretization of rainfall stations, and the effect of lumped parameter calculations.

Statistical analyses also quantitatively confirmed the good performance of monthly streamflow simulation with results of Ens = 0.76, Ens’ = 0.60 and *R*^2^ = 0.77 for calibration and Ens = 0.87, Ens’ = 0.65 and *R*^2^ = 0.94 for validation (Table 2).

These results for the calibration and validation periods demonstrated that this HSPF model provided a good representation of the monthly hydrologic processes in the watershed.

#### 3.1.3. Daily Streamflow Simulation Results and Model Performance

**Figure 5:**
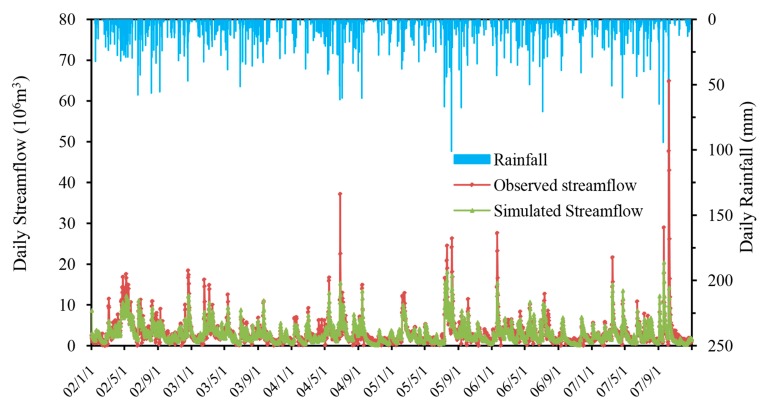
Comparison between simulated and observed monthly streamflow in the Xitiaoxi watershed during the calibration period (1 January 2002–31 December 2007).

**Figure 6:**
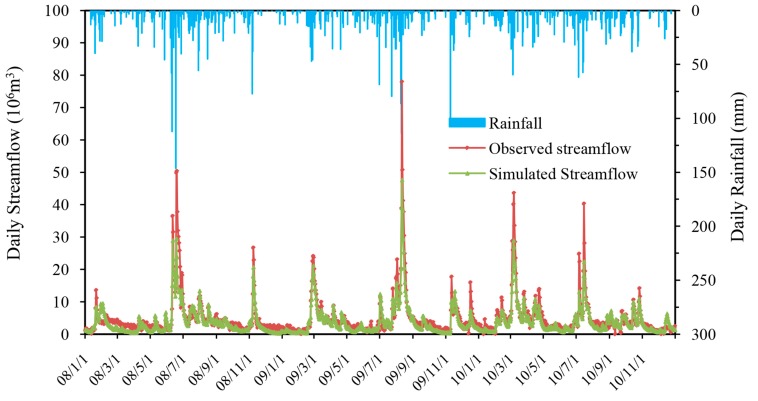
Comparison between simulated and observed daily streamflow in the Xitiaoxi watershed during the validation period (1 January 2008–31 December 2010).

As Figure 5 and Figure 6 show, the HSPF model also performed very well in predicting streamflow at daily time-steps. The simulated daily streamflow closely matched with observed values, and has a consistent tendency with rainfall. Even though, there is an amount of underestimation of simulated peak streamflow, especially in storm-event days. Statistical indicators also quantitatively confirm the above analyses. During the calibration period (1 January 2002–31 December 2007), the *R*^2^, Ens and Ens’ for daily streamflow simulation were 0.65, 0.65, and 0.48, and during the validation period (1 January 2008–31 December 2010), the *R*^2^, Ens and Ens’ were 0.86, 0.80 and 0.54, respectively. Compared with the results of annual and monthly time-steps, these indicators of model performance showed that the HSPF was satisfactory to simulate streamflow at daily time-steps.

In common, whether at annual or monthly and daily time-steps, the simulated streamflow in rainy days was more underestimated than observed values. This phenomenon may be caused by the small rainfall events before the storm events. When one small rainfall occurred before the storm event, it may replenish the soil moisture and made the hydrological model overemphasize the effect of soil moisture on the yield of runoff yield. Gallagher and Doherty [59] concluded that rainfall was more local in reality than that simulated by the model, and thus, localized storm events may also contribute the common performance. Diaz-Ramirez [51] suggested that the spatial resolution of rainfall gauges had an effect on storm runoff simulation. In this study, there are seven rainfall stations within the watershed, however, the effect of local rainstorms on runoff simulation still exists due to the spatial heterogeneity of rainfall in mountainous areas. Thus, more reasonable rainfall data with high spatial resolution be required for the future studies. 

The performance of hydrological simulations was evaluated at daily, monthly and annual time-steps, and although the model’s abilities were different, they all reached satisfactory, good and very good standards. In general, the HSPF model can properly capture the change processes of streamflow in annual, monthly and daily time steps.

### 3.2. Nutrients Simulation Results and Model Performance

**Figure 7:**
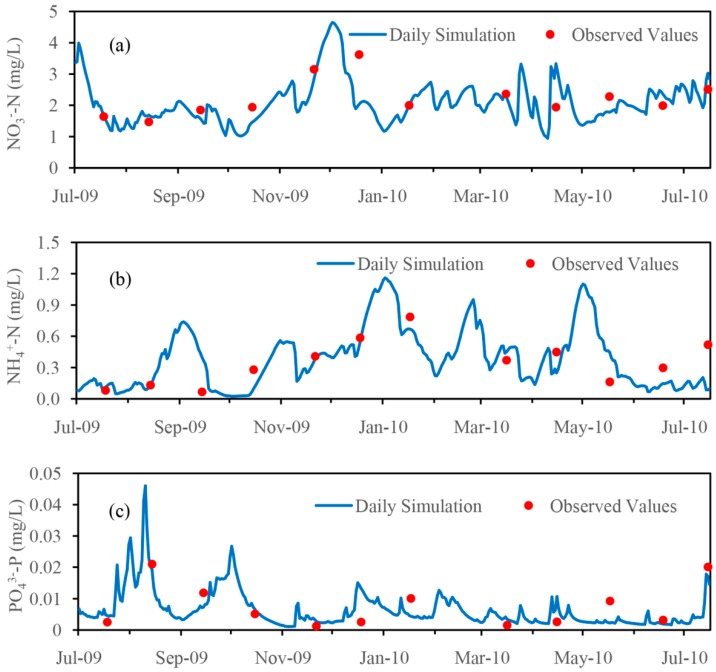
Comparison of the simulated and observed nutrient concentrations for the calibration at the Hengtang station (July 2009–July 2010). (**a**) NO_3_^−^-N; (**b**) NH_4_^+^-N; (**c**) PO_4_^3−^-P.

**Figure 8:**
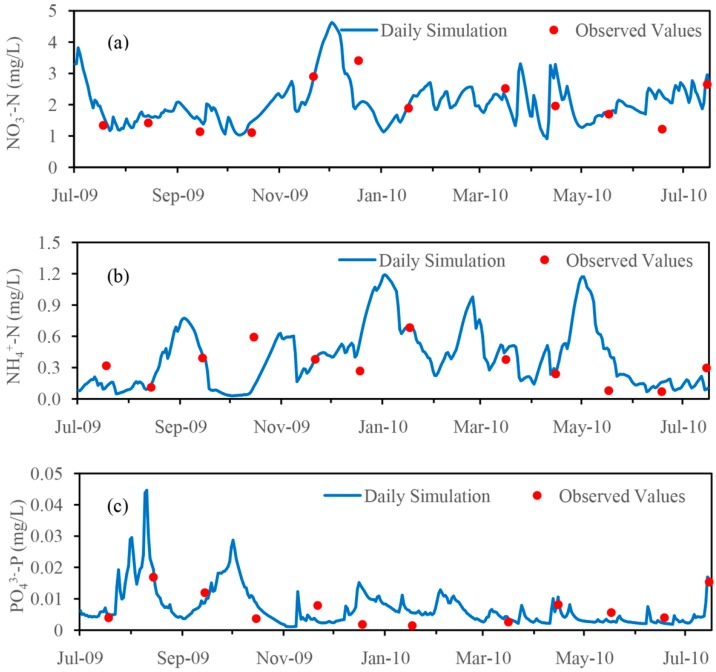
Comparison of the simulated and observed nutrient concentrations for the validation at the Ancheng Bridge station (July 2009–July 2010). (**a**) NO_3_^−^-N; (**b**) NH_4_^+^-N; (**c**) PO_4_^3−^-P.

For nutrient simulation, the model was also run for the period from 2002 to 2010. The observed monthly nutrient concentrations at the Hengtang station and Ancheng Bridge station from July 2009 to July 2010 were used to calibrate and validate the nutrient simulation. Figure 7 and Figure 8 show a comparison of the simulated and observed nutrients (nitrate, ammonium and orthophosphate) concentrations at the Hengtang and Ancheng Bridge stations for the calibration and validation, respectively.

As shown in Figure 7, at the calibration site of Hengtang station, the simulated concentrations for the three components all exhibited similar trends to observed values during the monitoring period from July 2009 to July 2010. The statistics of model performance were also carried out, and the results (Table 3) generally demonstrated satisfactory simulation for all nutrient components. The *R*^2^ for nitrate, ammonium and orthophosphate were 0.73, 0.82 and 0.92, respectively. The Ens and Ens’ for nitrate concentration showed good results at 0.67 and 0.45, respectively. Moreover, the PBIAS of the nitrate monthly mean concentration is just −4.00%, and this also showed good simulation in average condition even though there is a little underestimation. For ammonium simulation, the Ens and Ens’ for ammonium were 0.74 and 0.53, respectively, which is better than nitrate simulation, although the PBIAS of the mean concentration is 14.81% and shows some overestimation. The orthophosphate also showed satisfactory simulation results with Ens and Ens’ of 0.86 and 0.61, respectively, and the PBIAS of −11.38% indicated a little underestimation.

As for the validation of nutrient simulation at the Ancheng Bridge station, as Figure 8 shows, although there are some discrepancies, the simulations also give generally good results. The statistical analysis was performed on the model performance parameters, and the results show that the PBIAS between simulated and observed monthly mean concentration for nitrate, ammonium and orthophosphate were 5.37%, −2.28% and 18.31%, respectively. The *R*^2^ was 0.71, 0.58 and 0.76 for these three nutrients components, which are not as good as the calibration results. The Ens and Ens’ for nitrate simulation were 0.66 and 0.42, were 0.53 and 0.29 for ammonium simulation, and were 0.67 and 0.49 for orthophosphate simulation, respectively, and these evaluation parameters are slightly lower than the calibration results. In general, the results from model validation further confirmed the ability of the model to simulate monthly nutrient export with satisfactory model performance. These results for calibration and validation demonstrated that this HSPF model provided a reasonable representation of the monthly nutrient export processes in the watershed.

## 4. Discussion

The results of HSPF evaluation in this study show that the calibrated HSPF can well simulate the streamflow and nutrient transport in one mesoscale monsoon watershed. However, any model is not perfect, it is just a real-world approximation. Each model has its advantages and disadvantages in certain aspects and with specific applications [37]. In the application of the HSPF model, some issues should be considered carefully, such as the model input data, parameter values, model algorithms, observed field data, calibration accuracy, and so on [29].

HSPF model simulation requires abundant spatial and attribute data, especially land use data, a digital elevation model (DEM) and meteorological data. High-quality input data are essential for good performance of model simulation. Even though one result from the study of scale-dependency and sensitivity of hydrological estimations to different land use and elevation datasets showed that HSPF-estimated stream flows are not sensitive to scale and spatial resolution of the datasets [60], more studies displayed obvious effects of input datasets on hydrological, sediment and nutrient simulations [61,62,63,64,65]. The Geographic Information Retrieval and Analysis System (GIRAS), the Moderate Resolution Imaging Spectroradiometer land cover product (MODIS MOD12Q1) and the National Land Cover Dataset (NLCD), datasets were used to evaluate the impact of land use on hydrology and sediment components, and the results showed that sediment predictions were more sensitive than streamflow predictions to the scale and resolution of land use datasets [61,62]. Choosing the right land use dataset will impact the modeling of sediments and, potentially, other water quality constituents that are related to agricultural activities [61]. The hydrological and water quality parameters are also sensitive to a watershed’s topographic characteristics; for example, rugged topographic characteristics enabled the Wolf River Basin simulation to have better results [63]. The accuracy of streamflow predictions of the HSPF model is affected by sparse meteorological data. By design, precipitation and other meteorological data from weather stations serve as standard model inputs. In practice, these stations may be unable to capture the spatial heterogeneity of precipitation events, especially if they are few and far between [65]. With high spatial and temporal resolutions, the North American Land Data Assimilation System (NLDAS) data has the potential to improve stream flow predictions [64]. The modeling performance of HSPF increased when using the NASA-modified precipitation data, resulting in better streamflow statistics and, potentially, in improved water quality assessment [65]. This could also expand the potential use of BASINS and HSPF to parts of the world where good meteorological data are lacking [65]. In addition, it is also important to note that the more grid cells and precipitation values, the more reaches created and the more manual editing involved in large watersheds [65]. In this study, the land use data are derived from the artificial interpretation of remote sensing data, DEM produced from 1:50,000 relief maps, rainfall data collected from eight meteorological stations, which can represent the temporal and spatial differences in hilly and mountain zones with a monsoon climate. The good results of hydrological and water quality simulation in part depend on these high-quality input data.

HSPF simulation requires a large number of parameters, and parametric uncertainty is one problem for almost all models. Some parameters can interfere with each other during the calibration processes [4]. To obtain good simulation results, the model needs extensive calibration, and different combinations of parameters may yield the same simulation results. Therefore, it is important to understand and identify the key ones among the numerous parameters of the HSPF model. Mishra [27] evaluated 15 hydrological parameters using perturbation analysis, and Diaz-Ramirez [51] selected eight hydrological parameters using PEST to determine the sensitive parameters. Eighteen parameters were used for model calibration, and parameter uncertainty of the HSPF was explored using three methods by Iskra I and Droste [66]. Combined application of the Rosenblueth method and sensitivity analysis, and uncertainty propagation through the parameters and structure of the HSPF model was determined by Patil [67]. In this study, based on the HSPF technical guides and literature, 17, 15, 15, and 9 parameters were selected for the sensitivity analyses of flow, ammonia, nitrate and orthophosphate, respectively [40]. After investigating the sensitivity of these parameters using the differential sensitivity analysis (DSA) method, 21 key parameters were identified and then their values were calibrated (Table 1), and the result is beneficial to facilitate application of the HSPF in other similar watersheds or regions.

The application of the HSPF model was also affected by the lack of observed data, the complexity of the model structure and other factors. Disagreement between simulation and observed data might appear in modeling practice due to an insufficient observation dataset, model complexity and calibration technique [29]. Many cases show that the lack of observed data limits the performance of model simulation, especially for the nutrient simulations. Diaz-Ramirez et al. [68] reported the application of the HSPF model to simulate phosphorus exports from an agricultural watershed in Mississippi, where they calibrated the model for flow, sediment and phosphorus, but obtained poor performance for phosphorus during the monthly time-steps. Model outputs representing average values of one time step may differ from observed values of specific points of time [29]. In this study, water quality data were not sufficient and were collected at monthly intervals, and this does not reflect the continuous processes of water quality diurnal variation. Compared with previous studies of applying the HSPF model to simulate nutrients, the results of this study are still acceptable. As concluded by John et al. [69], better results can be achieved through the use of more detailed, complete and accurate data. With the increase in monitoring data and the degree of data openness in developing countries, this situation of lacking observed data will be solved. In addition, the HSPF model relies on many empirical relationships to express the physical process, and some of the equations or algorithms still have space to improve and perfect [70,71]. The HSPF instream model assumes that the receiving water body model is well-mixed with width and depth. For overland flows, the model assumes one-directional kinematic wave flow [37]. Due to the limitations of the model itself, some problems cannot be done by a particular model alone. Therefore, it is necessary to integrate HSPF with other models to carry out a comprehensive simulation in more complex watersheds [72,73,74,75].

## 5. Conclusions

The performance evaluation for hydrological and nutrient simulation using the HSPF in a mesoscale monsoon watershed was carried out in this paper. Streamflow simulation was calibrated from 1 January 2002 to 31 December 2007 and then validated from 1 January 2008 to 31 December 2010 using daily observed data at the Gangkou station, which is the outlet of the Xitiaoxi watershed. Nutrient simulation was calibrated and validated at the Hengtang station and the Ancheng Bridge station during July 2009 to July 2010 using monthly observed data. The evaluation of the model performances shows that the HSPF reached very good, good and satisfactory for the streamflow simulations at annual, monthly and daily time-steps, respectively. The determination coefficient (*R*^2^) was 0.87, 0.77 and 0.63, and the Nash-Sutcliffe coefficient of efficiency (Ens) was 0.82, 0.76 and 0.65 for the calibration results in these three time-steps, respectively. Although the observed data of nutrients are limited, the quantitative evaluation on the performance of nutrient simulation showed that HSPF achieved satisfactory results. The *R*^2^ was 0.73, 0.82 and 0.92, and the Ens was 0.67, 0.74 and 0.86 for nitrate, ammonium and orthophosphate in the calibration period, respectively. These evaluation results of model performance showed that the streamflow and nutrients were well simulated over the study period, and proved that the HSPF model could be successfully used to describe streamflow and nutrient transport in a mesoscale watershed located in the East Asian monsoon climate area. Additionally, issues such as the quality of input data, parameter choice, limited observed data and other uncertainty should be carefully considered when applying the HSPF model in watersheds. Furthermore, the presented parameters from this study could be useful for other watersheds that are under similar hydroclimatic conditions as the Xitiaoxi watershed.

Further studies are required to establish a parameter library and to assess the suitability of applying HSPF under different geographical conditions in variable spatial scales and time series. As a comprehensive hydrological and water quality model, the HSPF is lumped but sophisticated, and can present hydrological and water quality transport processes of a watershed and the results can be utilized in developing watershed management measures. With publicly available data such as DEMs and land use data becoming more accessible, the application of HSPF will become more extensive in developing countries. It is highly recommended to introduce the HSPF model into watershed water resources and environmental management, to discriminate nonpoint source pollution and nutrient biogeochemical cycling in different soils, and to evaluate the impacts of watershed management measures on hydrology and water quality under climate change and land use changes.

## Figures and Tables

**Table 1:** List of adjusted parameters for calibration of HSPF (Hydrological Simulation Program–Fortran) model.

Category	Parameter	Explanation	Unit	Original Value	Calibrated Value
Hydrological	LZSN	Lower zone nominal soil moisture storage	in	6	0.584–2.39
	INFILT	Index to infiltration capacity	in·h^−1^	0.16	0.30–10.90
	AGWRC	Base groundwater recession	day^−1^	0.98	0.949
	DEEPFR	Fraction of GW * inflow to deep recharge	-	0.1	0.35
	UZSN	Upper zone nominal soil moisture storage	in	1.128	0.22–2.56
	LZETP	Lower zone ET ^^^ parameter	-	0.1	0.50
	BASETP	Fraction of potential ET from baseflow	-	0.02	0.27
	CEPS	Initial interception storage	in	0.01	0.14
	UZS	Initial upper zone storage	in	0.3	3.62
Ammonia	MON-SQOLIM	Monthly values limiting storage of QUALOF ^※^	lb/ac	0.004–0.069	0.002–0.051
	MON-IFLW-CONC	Monthly concentration of QUAL ^#^ in interflow	qty/ft^3^	0.03–0.2	0.03–0.144
	MON-GRND-CONC	Monthly concentration of QUAL in active groundwater	qty/ft^3^	0.025–0.1	0.250–4.30
	KATM20	Unit oxidation rate of total ammonia at 20 °C	h^−1^	0.014	0.015
	MALGR	Maximal unit algal growth rate for phytoplankton	L·h^−1^	0.085	0.102
Nitrate	MON-SQOLIM	Monthly values limiting storage of QUALOF	lb/ac	0.09–3.16	0.09–3.16
	MON-IFLW-CONC	Monthly concentration of QUAL in interflow	qty/ft^3^	0.4–19	0.192–5.0
	MON-GRND-CONC	Monthly concentration of QUAL in active groundwater	qty/ft^3^	0.3–12	0.052–3.780
Phosphorus	MON-ACCUM	Monthly values of accumulation rate of QUALOF	lb/ac·day	0.003–0.012	0.003–0.050
	MON-IFLW-CONC	Monthly concentration of QUAL in interflow	qty/ft^3^	0.009–0.1	0.0009–0.15
	MON-GRND-CONC	Monthly concentration of QUAL in active groundwater	qty/ft^3^	0.005–0.05	0.0001–0.32
	MALGR	Maximal unit algal growth rate for phytoplankton	L·h^−1^	0.085	0.102

* GW: groundwater; ^^^ ET: evapotranspiration; ^※^ QUALOF: Quality associated with Overland Flow; and ^#^ QUAL: Water quality constituents, such as ammonia and nitrate.

**Table 2:** Calculated statistical parameters of model performance for streamflow calibration/validation.

Item	Calibration				Validation			
	*R*^2^	Ens	Ens’	PBIAS (%)	*R*^2^	Ens	Ens’	PBIAS (%)
Annual flow	0.87	0.82	0.56	−2.1	0.94	−19.65	−4.35	−15.3
Monthly flow	0.77	0.76	0.60	−2.1	0.94	0.87	0.65	−15.3
Daily flow	0.63	0.65	0.48	−2.1	0.86	0.80	0.54	−15.3

*R*^2^: the coefficient of determination, Ens: Nash-Sutcliffe coefficient of efficiency, Ens’: revised Ens, PBIAS: the percent bias.

**Table 3:** Statistical parameters of model performance for nutrient simulation.

Items	Calibration				Validation			
	*R*^2^	Ens	Ens’	PBIAS (%)	*R*^2^	Ens	Ens’	PBIAS (%)
NO_3_-N	0.73	0.67	0.45	−4.00	0.71	0.66	0.42	5.37
NH_4_-N	0.82	0.74	0.53	14.81	0.58	0.53	0.29	−2.28
PO_4_-P	0.92	0.86	0.61	−11.38	0.76	0.67	0.49	18.31
